# Supplementary material for: A biochemical mechanism for Stu2/XMAP215-family microtubule polymerases
Source: eLife. 2026 Sep 3;14:RP107818. doi: 10.7554/eLife.107818 (PMC13541299; doi:10.7554/eLife.107818)
Supplement: Peer review history

# Editor report: eLife Assessment

**DOI:** 10.7554/eLife.107818.3.sa0

**Authors:** Kassandra M Ori-McKenney

**Affiliations:** University of California, Davis United States

**Keywords:** Convincing, Important

In their **important** manuscript, Gangadharan, Kober and Rice focus on how Stu2/XMAP215-family microtubule polymerases use their TOG domains to catalytically promote microtubule growth, testing whether their mechanism follows an enzyme-like kinetic model similar to that of actin polymerases. The authors integrate measurements including microtubule polymerization rates and TOG-tubulin binding kinetics to **convincingly** show that Stu2 follows an enzyme-like model where tight tubulin binding enables efficient polymerization, revealing a shared mechanism with actin polymerases despite their evolutionary divergence. This work will be of general interest to the cell biology and biophysics communities.

# Referee report: Reviewer #1 (Public review)

**DOI:** 10.7554/eLife.107818.3.sa1

This study by Gangadharan and colleagues provides significant progress towards a quantitative biochemical mechanism for Stu2 polymerase activity. A key conceptual advance is the novel application of an enzyme-like model, initially developed for the actin polymerase Ena/VASP, to Stu2.

Strength:

New refined affinity measurements for a Stu2 TOG domain using Bio-layer interferometry show more than an order of magnitude higher affinity of TOG domains to tubulin compared to previously published reports.

The findings reinforce the "concentrating reactants" or, more specifically, for TOG-domain proteins, the "tubulin-shuttling antenna" model, compared to the "polarized unfurling" model, a more speculative structural hypothesis.

The manuscript builds upon a series of previous manuscripts that showcase the profound intellectual engagement with microtubule polymerization mechanisms by TOG-domain proteins from the Rice lab, a thought leader in microtubule polymerization for over a decade.

Minor weakness:

The affinity discrepancy is not fully resolved by side-by-side measurements, which seem to be not feasible as not all buffer conditions are compatible with all assays.

# Referee report: Reviewer #2 (Public review)

**DOI:** 10.7554/eLife.107818.3.sa2

Summary:

The manuscript from the Rice lab by Gangadharan et al., submitted to eLife, investigates the polymerization mechanism of the yeast microtubule polymerase Stu2. The lab has published a number of articles demonstrating the structural basis by which the two TOG domains of Stu2 each bind free tubulin heterodimers and has developed a tethered polymerization model by which the TOG domains drive polymerization by shuttling those tubulin subunits onto the microtubule plus end. A second model was proposed by Nithianantham et al. (eLife, 2018) based on a closed - to - open transitional state in which Stu2 unfurls and loads two longitudinal associated tubulin heterodimers onto the microtubule plus end. While the second model is not directly tested, the current work aims to further characterize/model the tethered polymerization model using a kinetic framework developed by developed by Breitsprecher et al. for Ena/VASP actin polymerization activity, using a model that is enzymatic (EMBO J., 2011). The general architecture and function of Ena/VASP on actin polymerization versus Stu2 on microtubule polymerization is a reasonable relation and hits upon, as the authors note, potential convergent mechanistic evolution across distinct cytoskeletal networks. The model effectively treats tubulin as the substrate, and the polymerized microtubule plus end as the product. If Stu2 is "enzymatic" in this framework, the model predicts it would behave with Michaelis-Menten kinetics, that there would a Vmax, and polymerase activity would either be "affinity limited" by TOG:tubulin affinity (KD) and/or "kinetically limited" by TOG:tubulin association (Kon) and transfer of tubulin to the microtubule plus end (Kt). The authors find that the Brietsprecher model works well for Stu2 activity, and that Stu2 best aligns with a "kinetically limited" model. The work is interesting and adds to the growing elucidation of the Stu2 microtubule polymerase model. While yeast microtubule polymerases are somewhat distinct in their architecture, there is significant overlap that findings from the manuscript can be utilized to inform the mechanisms of larger, more complex microtubule polymerases such as human ch-TOG.

Strengths:

The manuscript invokes the enzymatic model of Breitsprecher et al. used for Ena/VASP and conducts an elegant series of (mostly established) experiments to determine whether Stu2 microtubule polymerase activity aligns with the model - which they conclude does align, supported by the data/results obtained.

Weaknesses:

The authors used biolayer interferometry to measure TOG:tubulin affinity. The affinities obtained were significantly higher affinities than the lab obtained in an earlier publication using analytical ultracentrifugation. While differences in buffer and salt conditions may underlie these differences, additional runs using comparable buffer systems, or use of a third independent assay to measure affinities would have added rigor.

The discussion could be expanded to better compare and contrast the results with both existing polymerase models introduced in the introduction, as well as expanded to look at reversible enzymatic activity (microtubule depolymerization at low to zero tubulin concentrations) and microtubule plus versus minus end activity.

Comments on revised version.

The revised submission has addressed these comments adequately.

# Referee report: Reviewer #3 (Public review)

**DOI:** 10.7554/eLife.107818.3.sa3

Summary:

This study by Gangadharan and colleagues seeks to establish a quantitative biochemical model for the microtubule polymerase activity of Stu2. Stu2 is the budding yeast member of the XMAP215 protein family, which is broadly conserved across eukaryotes. XMAP215 proteins play a wide variety of important roles in cells, and these are attributes to effects on microtubule dynamics. Many studies over the last ~20 years have shown that XMA215 proteins selectively associate with microtubule ends where they increase rates of microtubule assembly and disassembly. More recently, structural biology and biochemical studies by the authors and other groups have shown that the multiple TOG domains on XMAP215 proteins are tubulin-binding domains that selectively bind to curved tubulin, which is present in solution and at microtubule ends, but not to straight tubulin which is present in the walls of the microtubule lattice. This has led to the general model that XMAP215 proteins promote polymerization by delivering soluble tubulin to the growing plus end, and two distinct models have been proposed to explain the mechanism. The 'concentrating reactants' model proposed previously by the authors suggests that TOG domains grab hold of tubulin in solution and concentrate at the microtubule end. The 'polarized unfurling' model proposed by the Al Bassam lab suggests that XMAP215 delivers multiple tubulins to the end, using a stepwise mechanism involving different roles for each TOG domain. The current study seeks to improve our understanding of the mechanism by developing a quantitative model to explain the binding and release of tubulins, the number of Stu2 molecules at the end, and the overall rate of tubulin addition. The authors accomplish this goal using new experimental data. The final model fills in new details of the mechanism. The authors draw a comparison between Stu2 and the actin polymerase which bears similarity to the Ena/VASP and suggest a convergent strategy for cytoskeletal polymerases.

Strengths:

This is a focused and clearly written study that incorporates prior knowledge of XMAP215 and draws inspiration from the actin field. The data are clear and convincing, and the study accomplishes its goal of generating a new, quantitative model for Stu2. The model will be important for microtubule researchers to predict and test key points for altering XMAP215 activity across different organisms and potentially for different tubulin substrates. The comparison to Ena/VASP may also inspire similar comparisons across other microtubule and actin regulators, which could lead to new insights across cytoskeletal fields.

Weaknesses:

The study is without major weaknesses.

# Author comment: Author response

**DOI:** 10.7554/eLife.107818.3.sa4

**Authors:** Binnu Gangadharan, Daniel L Kober, Luke M Rice

**Affiliations:** The University of Texas Southwestern Medical Center Dallas United States; University of Texas Southwestern Medical Center Dallas United States; The University of Texas Southwestern Medical Center Dallas United States

The following is the authors’ response to the original reviews.

**eLife Assessment**

In their important manuscript, Gangadharan, Kober and Rice focus on how Stu2/XMAP215-family microtubule polymerases use their TOG domains to catalytically promote microtubule growth, testing whether their mechanism follows an enzyme-like kinetic model similar to that of actin polymerases. The authors integrate measurements including microtubule polymerization rates and TOG-tubulin binding kinetics to convincingly show that Stu2 follows an enzyme-like model where tight tubulin binding enables efficient polymerization, revealing a shared mechanism with actin polymerases despite their evolutionary divergence. This work will be of general interest to the cell biology and biophysics communities.

Thank you for the favorable assessment of our manuscript.

**Public Reviews:**

**Reviewer #1 (Public review):**

This study by Gangadharan and colleagues provides significant progress towards a quantitative biochemical mechanism for Stu2 polymerase activity. A key conceptual advance is the novel application of an enzyme-like model, initially developed for the actin polymerase Ena/VASP, to Stu2.

New refined affinity measurements for a Stu2 TOG domain using Bio-layer interferometry show more than an order of magnitude higher affinity of TOG domains to tubulin compared to previously published reports.

The findings reinforce the "concentrating reactants" or, more specifically, for TOGdomain proteins, the "tubulin-shuttling antenna" model, compared to the "polarized unfurling" model, a more speculative structural hypothesis.

The manuscript builds upon a series of previous manuscripts that showcase the profound intellectual engagement with microtubule polymerization mechanisms by TOG-domain proteins from the Rice lab, a thought leader in microtubule polymerization for over a decade.

Minor remarks:

(1) A major new experimental finding of this paper is the affinity of TOG domains, which is more than an order of magnitude lower (10 nM) than previous measurements from the same lab (~200 nM). The authors attribute this change to ionic strength differences between buffer conditions, citing the lab's previous work (Ayaz et al., 2014). This argument left me contemplating what the buffer conditions are in both experiments, and I wonder if other readers would feel the same. After going down the rabbit hole, I believe the difference in ionic strength is ~2.3 fold, and at least on the back of my envelope, this works out beautifully with the measured differences in affinities. A short version of this argument may strengthen the manuscript.

This is a good comment. We should have been clearer about the different buffer conditions. The revised manuscript now explicitly states how the two buffers in question differ in pH and ionic strength. (Page 8, ‘Tubulin binds rapidly …’ section). We tried to perform comparative measurements of TOG:tubulin affinity in the two buffer systems using biolayer interferometry, then analytical ultracentrifugation and isothermal titration calorimetry, but in each technique one or the other buffer caused aggregation, nonspecific binding, or some other artifact that prevented such an analysis. This is stated in the revised manuscript Page 9, final paragraph before the ‘Unifying measurements …’ section. Along the lines of the reviewer’s ionic strength calculation, and consistent with the increase in affinity we observed with lower ionic strength, we now also state that prior measurements from the Al-Bassam lab (Nithianantham, 2018) showed that TOG:tubulin affinity decreases ~20-fold with higher ionic strength (100 mM KCl vs 200 mM KCl).

(2) I am wondering if there may be an alternative explanation to tubulin binding by TOG being the kinetically rate-limiting step for polymerase function:

TOG + Tubulin ⇌ TOG:Tubulin (fast binding rate, high-affinity binding)

TOG:Tubulin + MT_end → TOG:MT (tubulin is incorporated into MT, fast transfer rate)

The binding rate is 3/s, and the transfer rate is 5/s.

I was wondering if the following step should be considered, which involves a conformational change of tubulin (e.g., straightening) TOG:MT → TOG + MT (ratelimiting straightening and unbinding of TOG from the lattice).

This is an interesting thought that highlights a gap in the understanding of microtubule dynamics.

Presumably, the affinity of TOGs for straight tubulin is practically zero for the purpose of this discussion, as there is no lattice binding, which means unbinding is likely very rapid; however, straightening may be the rate-limiting factor here.

In theory, straightening should also be rapid; however, we lack measurements of how fast or slow this step occurs within the context of a TOG domain, which presumably skews the process towards curved tubulin.

We agree (based on prior observations) that the affinity of these TOGs for straight tubulin is negligeable in this context. There is much less data about the timescale of tubulin straightening, with or without a TOG domain bound, or even about how tubulin interactions with the microtubule end affect the balance of preferred conformations and/or the rate of conformational change. It’s an extremely interesting topic. Because the straightening process the reviewer envisions is zero-order, the transfer rate in our model could in principle reflect slow straightening (in this view the ‘delivery’ step would need to be very fast, i.e. not rate-limiting). Because there is so little data about this, and because there are not yet methods to study or perturb the timescale of straightening on the microtubule, we prefer not to engage too deeply. We added a sentence to acknowledge this alternative possibility in the revised manuscript (bottom of Page 4 and top of Page 5).

A hypothetical Stu2, when bound to the microtubule end and with the TOG domain not disengaged from tubulin, would not permit the processivity of that molecule or the binding of a new molecule.

To emphasize the importance of unbinding, when it is not efficient, as reported for the T238 mutant that results in Stu2 lattice binding (Geyer et al., 2018), the polymerase becomes inefficient.

The mechanism of polymerase processivity has not been conclusively determined (the Geyer et al. 2018 eLife paper took a step in that direction, though). The model used in this paper is only concerned with how many polymerases are at the microtubule end at steady-state (as opposed to how long a particular polymerase acts before dissociating), so while we appreciate and are interested in these questions, we think it would be better to leave them for future work.

**Reviewer #2 (Public review):**

Summary:

The manuscript from the Rice lab by Gangadharan et al. investigates the polymerization mechanism of the yeast microtubule polymerase Stu2. The lab has published a number of articles demonstrating the structural basis by which the two TOG domains of Stu2 each bind free tubulin heterodimers, and has developed a tethered polymerization model by which the TOG domains drive polymerization by shuttling those tubulin subunits onto the microtubule plus end. A second model was proposed by Nithianantham et al. (eLife, 2018) based on a closed-to-open transitional state in which Stu2 unfurls and loads two longitudinally associated tubulin heterodimers onto the microtubule plus end. While the second model is not directly tested, the current work aims to further characterize/model the tethered polymerization model using a kinetic framework developed by Breitsprecher et al. for Ena/VASP actin polymerization activity, using a model that is enzymatic (EMBO J., 2011). The general architecture and function of Ena/VASP on actin polymerization versus Stu2 on microtubule polymerization is a reasonable relation and hits upon, as the authors note, potential convergent mechanistic evolution across distinct cytoskeletal networks. The model effectively treats tubulin as the substrate, and the polymerized microtubule plus end as the product. If Stu2 is "enzymatic" in this framework, the model predicts it would behave with Michaelis-Menten kinetics, that there would a Vmax, and polymerase activity would either be "affinity limited" by TOG:tubulin affinity (KD) and/or "kinetically limited" by TOG:tubulin association (Kon) and transfer of tubulin to the microtubule plus end (Kt). The authors find that the Brietsprecher model works well for Stu2 activity, and that Stu2 best aligns with a "kinetically limited" model. The work is interesting and adds to the growing elucidation of the Stu2 microtubule polymerase model. While yeast microtubule polymerases are somewhat distinct in their architecture, there is significant overlap that findings from the manuscript can be utilized to inform the mechanisms of larger, more complex microtubule polymerases such as human ch-TOG.

Thank you for the nice summary and favorable comments.

Strengths:

The manuscript invokes the enzymatic model of Breitsprecher et al. used for Ena/VASP and conducts an elegant series of (mostly established) experiments to determine whether Stu2 microtubule polymerase activity aligns with the model, which they conclude does align, supported by the data/results obtained.

Weaknesses:

The authors used biolayer interferometry to measure TOG:tubulin affinity. The affinities obtained were significantly higher than the lab obtained in an earlier publication using analytical ultracentrifugation. While differences in buffer and salt conditions may underlie these differences, additional runs using comparable buffer systems, or the use of a third independent assay to measure affinities, would have added rigor.

This is a good question that was also raised by reviewer #1. We tried hard to perform comparative measurements of TOG2:tubulin affinity in the two buffer systems using biolayer interferometry, then analytical ultracentrifugation and isothermal titration calorimetry, but in each technique one or the other buffer caused aggregation, nonspecific binding, or some other artifact that prevented such an analysis. This is now stated in the revised manuscript (page 9, final paragraph before the ‘Unifying measurements …’ section). We also added text to state that the affinity of TOG:tubulin interactions have been independently shown to depend on ionic strength in a way that seems consistent with what we observed: prior data from the Al-Bassam lab (Nithianantham, 2018) showed that TOG:tubulin affinity decreases ~20-fold with increased ionic strength (100 mM KCl vs 200 mM KCl) (page 9, final paragraph before the ‘Unifying measurements …’ section).

The discussion could be expanded to better compare and contrast the results with both existing polymerase models introduced in the introduction, as well as expanded to look at reversible enzymatic activity (microtubule depolymerization at low to zero tubulin concentrations) and microtubule plus versus minus end activity.

Thank you for the push to be more explicit about the two contrasting models. We made small changes to the introduction (top paragraph on page 3) and added a paragraph to the discussion to be clearer about how the existing models are or are not consistent with the present results (page 12, penultimate paragraph of the main text).

The ‘transfer’ reaction is treated as irreversible (analogous to catalysis by an enzyme), so the biochemical model we use for the polymerase cannot account for polymeraseinduced microtubule depolymerization at low to zero tubulin concentration. We added text to state that the model is limited to the growth reaction (page 4, last paragraph) but otherwise prefer to not engage too deeply in questions about the reverse reaction.

These polymerases are thought to be plus-end specific because of the domain organization of the protein: TOGs bind tubulin such that the N- to C-terminal polarity of the TOG corresponds to the plus- to minus-end polarity of the tubulin, and the basic region used to make a ‘slippery’ connection to the microtubule is located C-terminal to the TOGs. These two factors mean that it is only at the plus-end that TOGs can engage αβ-tubulins with the basic region contacting surfaces ‘deeper’ in the polymer. We added text about these issues, citing prior work, to the legend of Figure 5 (page 11). We chose to not elaborate much since it is not the primary focus of the paper.

**Reviewer #3 (Public review):**

Summary:

This study by Gangadharan and colleagues seeks to establish a quantitative biochemical model for the microtubule polymerase activity of Stu2. Stu2 is the budding yeast member of the XMAP215 protein family, which is broadly conserved across eukaryotes. XMAP215 proteins play a wide variety of important roles in cells, and these are attributed to effects on microtubule dynamics. Many studies over the last ~20 years have shown that XMA215 proteins selectively associate with microtubule ends, where they increase rates of microtubule assembly and disassembly. More recently, structural biology and biochemical studies by the authors and other groups have shown that the multiple TOG domains on XMAP215 proteins are tubulin-binding domains that selectively bind to curved tubulin, which is present in solution and at microtubule ends, but not to straight tubulin which is present in the walls of the microtubule lattice. This has led to the general model that XMAP215 proteins promote polymerization by delivering soluble tubulin to the growing plus end, and two distinct models have been proposed to explain the mechanism. The 'concentrating reactants' model proposed previously by the authors suggests that TOG domains grab hold of tubulin in solution and concentrate at the microtubule end. The 'polarized unfurling' model proposed by the Al Bassam lab suggests that XMAP215 delivers multiple tubulins to the end, using a step-wise mechanism involving different roles for each TOG domain. The current study seeks to improve our understanding of the mechanism by developing a quantitative model to explain the binding and release of tubulins, the number of Stu2 molecules at the end, and the overall rate of tubulin addition. The authors accomplish this goal using new experimental data. The final model fills in new details of the mechanism. The authors draw a comparison between Stu2 and the actin polymerase, which bears similarity to Ena/VASP, and suggest a convergent strategy for cytoskeletal polymerases.

Thank you for the good summary and favorable comments.

Strengths:

This is a focused and clearly written study that incorporates prior knowledge of XMAP215 and draws inspiration from the actin field. The data are clear and convincing, and the study accomplishes its goal of generating a new, quantitative model for Stu2. The model will be important for microtubule researchers to predict and test key points for altering XMAP215 activity across different organisms and potentially for different tubulin substrates. The comparison to Ena/VASP may also inspire similar comparisons across other microtubule and actin regulators, which could lead to new insights across the cytoskeletal fields.

Thank you for these comments.

Weaknesses:

The study is without major weaknesses, but there are several minor weaknesses worth noting. One is that the final model provides new details regarding the Stu2 mechanism, but does not provide a major new advance in our understanding of how the polymerase works. For example, the discussion does not clearly argue for whether the new results and model rule out either of the prior models. This appears consistent with the 'concentrating reactants' model, but does it clearly rule out the 'polarized unfurling' model?

Thank you for pointing out what in retrospect was an obvious ‘loose end’ in our discussion. The other referees raised the same point. We made small changes to the introduction and added a paragraph to the discussion to be clearer about how the existing models are or are not consistent with the present results (top paragraph of page 3 and new penultimate paragraph of the manuscript on page 12).

A second minor weakness is that the comparison to Ena/VASP is not developed at a deep level based on the final model. I found these ideas exciting and want more critical consideration here, but perhaps it is better suited for a commentary piece to follow.

We appreciate the enthusiasm and understand where this comment is coming from. Because there has been a fair amount of recent movement in the understanding of TOG domains and what they can do, and because some of the mechanistically interesting parallels entail speculation, we agree with the referee’s suggestion that a future commentary will provide a better venue.

**Recommendations for the authors:**

**Reviewer #1 (Recommendations for the authors):**

Other minor remarks:

(1) Figure 2 C is missing the label for what should probably be TOG1*-TOG2.

Fixed

(2) Figure 5, lower left, is oddly cropped, showing residuals that are slightly distracting from the beauty of the model.

Apologies that the figure did not look good in the initial submission. We adjusted it and it looks much better in the revised submission.

(3) The dynamics assay buffer composition stated in the protein purification Method section is not the same as the PEM buffer used for the dynamics assay. And both are different from BRB80, which, with the chambers, are rinsed. This may very well be accurate, but it raises the question of why not stick to one version, as they are virtually the same.

Thanks for asking these questions, and sorry for the confusion. First, we should have used different names for the (barely) different buffers. This has now been corrected. Second, the PIPES concentration was not 90 mM, it was 100 mM as in our prior work and this discrepancy failed to get caught in proofreading. Why the other small differences? It’s a good question. The differences reflect an arbitrary decision made at the beginning of the work, there is not a deeper rationale.

(4) Out of curiosity: Why 90 mM PIPES and not 80?

Why not 80 mM PIPES? This is just a historical difference. The early measurements of yeast microtubule dynamics (e.g. Gupta … Himes MBoC 2002 and Bode … Himes, EMBO Rep 2003) that partly inspired us to use yeast as a model system used 100 mM PIPES as the working concentration, and we never deviated from that.

(5) Please state the source of PIPES.

Sorry for the oversight, we have added the source of PIPES (it is Millipore Sigma P6757).

**Reviewer #2 (Recommendations for the authors):**

(1) Page 4, last paragraph, the authors call out "Fig 1C" which I believe should be "Fig 1D".

We fixed this, thank for catching this error

(2) Figure 2C: The authors subtract basal tubulin polymerization (growth rate) from the rates measured in the presence of Stu2 constructs. One assumption in doing this is that Stu2 polymerization activity does not compete for the ability of tubulin (not bound to Stu2) to polymerize on the plus end. I think this is a logical assumption, but it would be beneficial for the authors to state this assumption.

We said this explicitly in the revised submission (first full paragraph on page 7), borrowing from the reviewer’s phrasing.

(3) Figure 2C: the label for the last bar is missing - presumably: " Stu2 (TOG1*-TOG2)".

Fixed.

(4) Figure 2D: Many of the KM values determined are at the border for points measured, or in one case, beyond the concentration of tubulin sampled. In this regard, the authors should discuss how well the fitted curves correlated with their data. Also, as Vmax and KM are calculated, it would be beneficial if another panel were produced (e.g., Figure 2E) in which the data were presented as a Lineweaver-Burk plot. Doing so, the authors would be able to test their enzymatic model by doping the system with their nonpolymerizable tubulin mutants, which should yield competitive inhibitor behavior but not change Vmax.

Thanks for pointing this out, we should have been more explicit about this point. We incorporated into the results section an explicit statement about this limitation (first full paragraph on page 7). The suggestion to use blocked mutants and Lineweaver-Burke plots is an interesting one that we hope to pursue in future work using blocked or other mechanism-specific mutants. But we think to do so is complicated enough to be beyond the scope of the present study.

(5) Figure 3: The authors quantitate the amount of Stu2-GFP fluorescence at microtubule plus ends using line scan analysis of the kymograph. Since the kymographs are processed images, it is more appropriate to integrate intensity from the original frames collected using a circular area. i.e., ID points on the kymograph, and return to the respective position in the corresponding frame to calculate background-subtracted GFP intensity at the plus end.

This is a fair point. We chose to stick with the kymographbased analysis because the symmetry of the point-spread function and lack of rapid variation in GFP and/or background intensity means that the kymograph analysis is adequate for the intensity-based comparison we were doing.

(6) Page 7, last line, the authors call out "Fig 2D" which I believe should be "Fig 1D".

Sorry for the error, we have corrected it.

(7) Figure 4A: The authors discuss the "sortase epitope," but technically, an epitope is the binding site specifically for an antibody, not to be used in general terms for proteinprotein interaction sites. As such, the authors should describe this as the "sortase recognition sequence" or something similar.

Thank you for noticing this; we had indeed used ‘sortase recognition sequence’ elsewhere in the paper but we did not catch this instance during proofreading. We have now used that same language in the legend for Fig. 4.

(8) Page 8, the authors state "KM is approximately equal to Kt/Kon and KM negligeable," but I think they mean "...and KD negligeable".

Thank you for noticing this typo. We corrected it.

(9) Page 9, first paragraph last sentence: the readership would be aided by modifying the sentence as follows (adding "Kon" and "Kt"): " ... must be kinetically limited by either the rate of TOG:tubulin binding (Kon) or by the rate of TOG-mediated transfer to tubulin to the microtubule end (kt)."

Very good suggestion, we implemented it.

(10) Page 9, second paragraph, the authors call out "Fig 2D", but perhaps they intended to call out "Fig 1D"?

Sorry for the error, the reviewer is correct and we fixed this.

(11) Page 9, second paragraph: The authors mention that the transfer rate of tubulin to the plus end via a TOG domain is close to the transfer rate of free tubulin to the growing plus end. Can the authors expand on why they are mentioning this comparison?

Thanks for the push to be clearer about this. The basic idea is that each ‘delivering’ TOG contributes 50% of the background (uncatalyzed) polymerization rate. So the presence of multiple TOGs (in a single polymerase or from multiple end-resident polymerases) can substantially increase the rate of polymerization. We added brief text to try to make this clearer (first paragraph on page 10).

(12) Page 9, second paragraph: "TOG-TOG2 polymerases" would be better phrased as "TOG2-TOG2 dimeric polymerases". Noting as well that "2" is missing from the first "TOG".

This is indeed better phrasing and we have adopted it (also corrected the missing ‘2’) (first paragraph on page 10).

(13) Page 13, BLI methods: The authors should list the final pH for the PIPES buffer (was it pH 6.9 as in the polymerization assay?).

Sorry for the oversight, we have added the pH and it was indeed 6.9.

(14) Page 13, BLI methods: What is "LR1-457"?

LR1-457 is lab-notebook-speak that did not get purged in editing; it refers to the polymerization-blocked tubulin mutant that also carried a sortase recognition sequence. We replaced ‘LR1-457’ with more evocative phrasing and corrected another typo we found there.

(15) In Ayaz et al. (eLife, 2014) Stu2 TOG1 and TOG2 affinities for tubulin were measured using AUC, for which the fitted curves appeared to correlate with the data quite well. As the authors note, the values were KD = 70 nM and 160 nM, respectively. This contrasts with the BLI measurement for TOG2-tubulin (~10 nM), which suggests that at least one of the experiments was off the mark - or, as the authors do note, that different buffer and salt condition was used could account for the differences, but that the BLI conditions align with the polymerization conditions (though not exactly) and thus are more appropriate to use. In a supplemental discussion, the authors should run the AUC values through the equation for their model and state what types of differences these values could imply for Stu2 mechanism. If the differences are significant for the Stu2 model derived, the authors should give thought as to whether a third assay should be employed to determine TOG-tubulin affinity. Based on the BLI reagents, it appears the authors would be well-positioned to conduct an assay using SPR. As a potential alternative, the authors could repeat the BLI experiment using the buffer conditions from the Ayaz et al., AUC work (25 mM Tris pH 7.5, 1 mM MgCl2, 1 mM EGTA, 100 mM NaCl, 20 μM GTP) - noting that BSA and Triton X-100 may need to be added as well. If the authors are able to replicate the ~160 nM affinity for TOG2:tubulin, this would be a reasonable way to bootstrap to the conclusion that the BLI is measuring affinity correctly and that the current PIPES-based BLI experiments yielded accurate data.

We tried hard to perform comparative measurements of TOG2:tubulin affinity in the two buffer systems. Unexpected challenges and personnel turnover made this slower than anticipated. The reviewer’s suggestions are completely reasonable, but ultimately it was not possible for us to get side-by-side results for TOG:tubulin affinity using the same measurement technique, whether it was biolayer interferometry, analytical ultracentrifugation, or isothermal titration calorimetry. For each technique one or the other buffer caused aggregation, non-specific binding, or some other artifact that prevented analysis. The fact that we were unable to compare the buffer conditions in this way is stated in the revised manuscript (page 9, last paragraph before the ‘Unifying measurements …’ section). We also added text to state that the affinity of TOG:tubulin interactions have been shown to depend on ionic strength in a way that is consistent with the changes we observed: prior data from the Al-Bassam lab (Nithianantham, 2018) showed that TOG:tubulin affinity decreases ~20-fold with increased ionic strength (100 mM KCl vs 200 mM KCl) (page 9, last paragraph before the ‘Unifying measurements …’ section). We also added some text to address the comment about affinity and whether/when the shuttle model would hold (first paragraph on page 11).

(16) A sentence or two in the discussion, relating how their data aligns (or not) with the Nithiantham model would be beneficial, especially as discussing the two models in the introduction was a central point.

We completely agree and have now added a paragraph to the discussion to explicitly address the two models and how are or are not supported by the new model and observations (page 12, penultimate paragraph of the main text).

(17) Discussion: The model in Figure 5 depicts Stu2 engaged with the microtubule, perhaps using its basic linker region (?). The authors could note this in the figure caption for 5A. The authors do not discuss the basis for plus-end polymerization activity versus polymerization activity at both the plus and minus ends. Do the authors propose that this is due to differential Kt values for the two ends and/or differential localization via the basic region to the two ends?

Thanks for bring this up. The plus-end selectivity of these polymerases is thought to result from the polarity of TOG:tubulin engagement and the positioning of TOG domains relative to the basic region that provides ‘slippery’ binding to the microtubule lattice. We have partially addressed these issues in the legend to Figure 5 (page 11).

(18) Brouhard (Cell, 2008) demonstrated that XMAP215 can catalyze the depolymerization of GMPCPP microtubules when no free tubulin, or very low levels of free tubulin, are present. This is interesting in that it indicates that the enzymatic activity is reversible. Can the authors comment on how their model would behave in the low-tozero free tubulin concentration regime? Would a different model have to be invoked?

This is an interesting comment. Because the enzyme-like model treats the transfer step as irreversible, the model cannot account for the kind of ‘depolymerase’ activity Brouhard and others have noted. A more general model that could also encompass the depolymerase activity at low-to-no free tubulin would need to explicitly model the step(s) involved in microtubule association and dissociation. These steps remain a major open question in the field and while it would be quite interesting, trying to address this in a model is beyond the scope of what we can confidently do given the data we have. To be more explicit about this assumption/limitation, we now point this out in the results section where the model is introduced (page 4, last paragraph).

**Reviewer #3 (Recommendations for the authors):**

(1) Figure 1D, legend. "...and a transfer rate constant kf that describes how fast...". Should kf be replaced with kt?

We made this correction, thanks for pointing the problem out

(2) Figure 2C. The x-axis label under the blue bar is missing. Also, I find the arrows to the left of the bars confusing and unnecessary.

We fixed the legend problem. We sympathize with the dislike of the arrows but respectfully prefer to keep them in the hopes of avoiding confusion about the fact we are fitting ‘growth rate attributable to Stu2’, not simply growth rate. The figure legend has been expanded to hopefully smooth this over.

(3) Figure 3. The kymographs are convincing, but it may be helpful for future studies to state here what the polymerization rates are for 0.6 µM and 1.4 µM yeast tubulin. These values are probably different than what one might expect for mammalian tubulin at those concentrations, and the authors could simply determine them from the slopes in the kymographs.

Good suggestion, we added the growth rates to the legend (as the reviewer expected, they differ from expectations based on mammalian tubulin).

(4) Results, page 9, line 11: "...yielded a value of 9.6 nM...". Should this be 8.9 nM, which is that value stated in Figure 4C?

Actually, these different values are correct. We just wanted to point out that whether we used response amplitudes or measured on- and off-rates, we get very similar values for K_D_. We changed wording to hopefully make this clearer: “Calculating the dissociation constant K_D_ from the measured rate constants (*K_D_* = *k_off_*/*k_on_*) instead of from the amplitudes yielded a value of 9.6 nM, in good agreement with the amplitude-based determination of 8.9 nM.” (page 9).
